# National Survey of Telemedicine Curricula Among Emergency Medicine Residencies

**DOI:** 10.5811/westjem.52946

**Published:** 2026-05-03

**Authors:** Christopher Reisig, Destinee Soubannarath Gwee, William Simmons, Brendan Tarantino, Neel Naik

**Affiliations:** * NewYork-Presbyterian Weill Cornell Medicine, Department of Emergency Medicine, New York, New York; † Saint Joseph Medical Center, Department of Emergency Medicine, Joliet, Illinois

## Abstract

**Introduction:**

Telehealth continues to reshape healthcare delivery in the United States. Recognizing its growing importance and the need for advances in education, the Association of American Medical Colleges released telehealth competencies in 2021, and the Accreditation Council of Graduate Medical Education (ACGME) recently proposed a structured telemedicine experience as part of all emergency medicine (EM) residencies. Despite these efforts, it is unclear whether EM residencies have adopted these new educational mandates. Our primary objective in this study was to understand whether (and how) U.S. EM residencies have implemented telehealth education.

**Methods:**

We developed a cross-sectional, national survey to describe existing telehealth curricula among ACGME-accredited EM residencies. Program directors were surveyed via email. Our primary outcome measure was the percentage of residencies with existing telehealth curricula. Secondary outcomes assessed telehealth curricula emphases, implementation barriers, and telehealth’s perceived importance to EM training.

**Results:**

Of 282 U.S.-based EM residencies, 67 programs responded (24% response rate). Of these, only five (7.5%) reported having a formal telehealth curriculum. Programs with curricula were likely to teach real-time telehealth skills (80%) and focus on data collection (80%), patient safety (80%), and communication (60%). Programs without curricula identified prioritization of other curricula (76%), insufficient faculty expertise (65%), and limited infrastructure (50%) as barriers. We also found that 61% of programs viewed telehealth education as of limited importance to EM training. At the same time, program directors expressed interest in the development of asynchronous telehealth content from trusted national EM organizations (60%).

**Conclusion:**

Formal telehealth curricula remain the exception rather than the rule among U.S. EM residencies. Despite accreditation bodies urging its adoption, telehealth education faces multiple barriers, including limited faculty expertise, lack of telehealth infrastructure, and low perceived education importance. Our research suggests that national organizations may play a key role in providing early telehealth education while programs adapt to these new educational requirements

## INTRODUCTION

In the evolving healthcare landscape, the integration of telehealth is reshaping medical care.^1^ Understanding how emergency medicine (EM) training programs respond to these changes is crucial. In 2021, the Association of American Medical Colleges (AAMC) established guidelines on telehealth competencies, providing a framework for their inclusion in medical schools and residency programs.^2^,^3^ More recently, the Accreditation Council of Graduate Medical Education (ACGME) proposed that all EM residents have a “structured experience in telemedicine” as part of their postgraduate training.^4^ Despite these guidelines and proposed residency requirements, it remains unclear whether telehealth has yet been integrated into EM curricula and whether programs are in a position to adopt these changes.

Social constructivist learning theory has long recognized the importance of the learning environment to any educational endeavor. We developed a cross-sectional survey for distribution to U.S. EM residency program directors in the belief it would provide insight into the learning environment in which telehealth curricula would be situated. Conceived before the recently proposed ACGME changes, our survey was designed to explore whether (and how) EM residencies in the U.S. have incorporated telehealth education into their curricula. We sought to understand what structural and educational barriers hinder implementation, including program directors’ perception of the value of telehealth to EM training, residency programs’ infrastructural capacity to provide telehealth, their faculty expertise to devise and execute a curriculum, and their hopes for supplemental educational support.

As telehealth continues to grow, it is vital that the next generation of emergency clinicians be adept at delivering digital healthcare. Our research provides a snapshot of the current educational landscape and the future work to be done.

## METHODS

We conducted an extensive literature review but found no validated surveys assessing the current state of telehealth education among EM residencies. We then designed a cross-sectional survey to assess the prevalence of telehealth education in EM residency training, identify which of the AAMC competencies were addressed by the telehealth curricula that did exist, and identify barriers to implementation. Consistent with best practices, we developed our survey’s construct by assembling panels of content experts in the respective fields of telehealth and medical education and collaboratively formulating the survey questions.^5^

The initial draft underwent a refinement process through cognitive interviews conducted with content experts in both fields. Incorporating insights from these interviews, we iteratively revised the survey and piloted it with content experts outside our institution whose feedback further refined the survey construct. The final survey (see Supplemental Section) was imported into a secure web-based survey platform (Qualtrics International, Inc, Provo, UT) and consisted of 15–17 questions depending on respondents’ answers to branch-logic questions. Most questions took the form of multiple-choice selection and nominal categories. Narrative boxes were also provided to capture data outside the provided choices. As part of the survey design, all respondents were asked (but not required) to identify their program to avoid duplicative responses in the final dataset. The survey and its associated consent language was approved by our institutional review board.

**Population Health Research Capsule**

What do we already know about this issue?

*Multiple governing medical bodies have stated telehealth education is critical to future physicians’ clinical training*.

What was the research question?

*To what extent have U.S. emergency medicine residencies incorporated telehealth education into their curricula, and what barriers exist to implementation?*

What was the major finding of the study?

*Very few EM residencies have telehealth curricula or the internal capacity to develop one*.

How does this improve population health?

*Our findings suggest there is emerging need for telehealth curricular development to meet the evolving field of healthcare*.

Our population of interest was ACGME-accredited, U.S.-based, categorical EM residency programs, of which 282 were listed on the Electronic Residency Application Service website as of September 2023. To reach this cohort, we directly emailed program directors. In that email, we asked them to respond to the survey or re-direct it to the member of their leadership team with the most knowledge regarding telehealth education. All communications included approved consent language, and respondents were informed that participation was purely voluntary and uncompensated. Initial emails were sent in September 2023, followed by two reminder emails sent to those program directors who (based on review of received surveys) had not responded to the initial email.

Responses were initially sorted by program name to screen for duplicates. Four programs chose to remain anonymous but completed the survey. We found sufficient heterogeneity in their responses to suggest these responses represented distinct programs rather than duplicate surveys, and all were included in the final data analysis. One program director (who disclosed the identity of their program) responded twice. There were no discrepancies between the two responses except that one survey contained additional information in the form of free text; we included the more complete survey for data analysis.

Survey results were imported from Qualtrics into R v4.4.0 (The R Foundation for Statistical Computing, Vienna, Austria). We formatted and tabulated the results using the R package gtsummary, with counts and percentages presented in tables. Some program directors declined to answer a particular question. In these instances, we calculated relevant counts and percentages based on provided responses, with the denominator reflecting the subset of those who responded. For free-text responses, we performed a thematic analysis to identify common narrative themes.

## RESULTS

### Overview of Programs Surveyed

Of the 282 programs emailed, 67 distinct programs responded for a response rate of 24% (67/282). Among respondents, there was good representation with respect to residency length (three vs four years), residency type (eg, university based, community based, etc.), community demographic served (eg, rural, urban, suburban), and regional location (Northeast, Midwest, South, etc). Of note, 25% (17/67) of surveyed program directors reported that their departments offered clinical services via telehealth; 69% (46/67) stated they did not offer any telehealth care; and 6% (4/67) did not answer. An overview of characteristics of the programs surveyed is described in Table 1.

Of the 67 residency program directors who responded, 7.5% (5/67) reported they had a formal telehealth curriculum. These five programs identified as being university based or affiliated and were in either the Northeast or Midwest. Four of the five (80%) were three-year programs. Of those five, one program director noted that while the residency had a telehealth curriculum, they did not provide clinical telehealth care. By contrast, 21% (13/62) of respondents reported offering clinical telehealth services but not having a formal telehealth curriculum.

### Characteristics of Telehealth Curricula

In those programs where a telehealth curriculum existed, we sought to better understand its broad characteristics. We asked about the forms of telehealth incorporated into the curriculum (eg, real-time patient care, physician-to-physician consultation, use of asynchronous medical data portals), the AAMC competencies addressed, and the educational modalities used. We also queried programs directors about their remaining educational needs with respect to telehealth. Characteristics of residency programs’ telehealth curricula are summarized in Table 2.

Of the five programs with a telehealth curriculum, 80% (4/5) taught real-time (or live patient care) telehealth; as noted earlier, one program did not provide clinical telehealth services. Two of the five programs (40%) taught via physician-to-physician consultations and mobile health technologies (eg, third-party smartphone apps). One program (20%) additionally used remote patient-monitoring training as part of its telehealth curriculum.

Of the six AAMC telehealth competencies, the most frequently addressed in telehealth curricula were “Data Collection and Assessment’ (80%, 4/5) and “Patient Safety and Appropriate Use of Telehealth” (80%, 4/5). “Communication via Telehealth” (60%, 3/5), “Ethical Practices and Legal Requirements” (60%, 3/5), and “Technology for Telehealth” (60%, 3/5) were the next most frequently taught; 40% (2/5) of programs addressed “Access and Equity” as part of their telehealth curricula. Most programs with telehealth curricula delivered their curricula via live lectures (80%, 4/5) and through a combination of mandatory and elective telehealth shifts (80%, 4/5). Two programs (40%, 2/5) additionally employed simulated telehealth experiences. Of note, one program used only mandatory shifts in their curricular delivery.

We also asked program directors whether their telehealth curricula might benefit from additional, external educational support. We framed the question in terms of the AAMC competencies, asking which (if any) of these domains could they better teach with external education content; we also asked how they would prefer to have that content delivered. Four of the five (80%) responded that their programs would benefit from additional resources addressing “Ethical Practices and Legal Requirements,” while 60% (3/5) noted additional support in teaching “Communication via Telehealth.” Educational support for each of the remaining competencies was endorsed by 60% (3/5) of program directors. In terms of educational modalities, program directors primarily expressed interest in simulation (60%, 3/6); live lectures (40%, 2/5); and asynchronous content created by a national EM organization (40%, 2/5). One program director responded that their telehealth curriculum was self-sufficient from an educational delivery standpoint.

### Challenges to Telehealth Curricula

If a program director reported not having a telehealth curriculum, we sought to better understand why. We asked about barriers to curricular implementation, the perceived importance of specific AAMC competencies, and how external educational support might augment any future telehealth curricula. A summary of these findings can be found in Table 3.

When asked what factors have prevented the implementation of a telehealth curriculum, the largest proportion of program directors (76%, 47/62) responded that they did not consider telehealth education to be a curricular priority. Additionally, 40% (25/62) of responding directors did not realize that AAMC telehealth competencies existed. Most reported insufficient faculty expertise to develop and run a curriculum (61%, 38/62 and 65%, 40/62, respectively), and 50% (31/62) indicated a lack of institutional infrastructure to support a curriculum. Of note, only 3.2% (2/62) of respondents reported that their department’s lack of clinical telehealth services served as a barrier to a telehealth curriculum.

We also sought to better understand how programs without telehealth education might envision a future curriculum. When asked what AAMC competencies they would prioritize, programs felt most strongly about teaching “Patient Safety and Appropriate Use of Telehealth” (65%, 40/62). There was, however, broad support for most of the competencies, with 58% (36/62) prioritizing “Communication via Telehealth,” 52% (32/62) prioritizing “Ethical and Legal Requirements,” and 50% (31/62) prioritizing “Data Collection and Assessment.” Of all the competencies, the least prioritized was “Technology for Telehealth” (31%, 19/62).

When asked what resources/modalities would best help them deliver a future telehealth curriculum, the most preferred option was asynchronous content created by a trusted national EM organization (60%, 37/62). There was relatively comparable interest in a variety of other modalities, including simulation (39%, 24/62), elective shifts (37%, 23/62), live lectures (27%, 17/62) and asynchronous content created by their home institution (26%, 15/62).

### Attitudes Toward Telehealth

**Figure 1:**
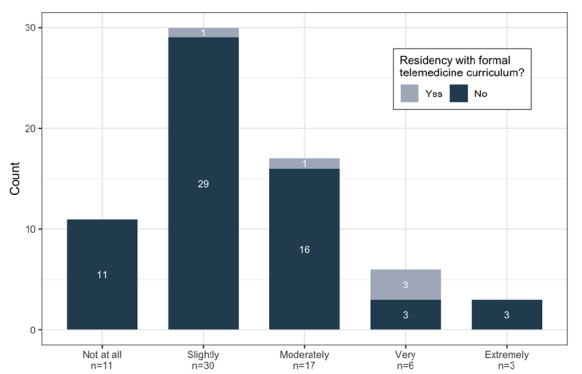
Emergency medicine program directors’ attitudes regarding the importance of a telehealth curriculum derived from a survey of 67 respondents.

Recognizing that multiple-choice questions might miss important qualitative information driving programs’ decisions to institute a telehealth curriculum, we also included free-text sections in the survey. We asked program directors, “In your opinion, how important will telehealth education be to EM residency training in the future?” To this last question, 38% (26/67) responded that telehealth education would be “moderately,” “very,” or “extremely” important, while 61% (41/67) considered telehealth education to be “slightly” or “not at all” important (Figure 1).

These broad sentiments were reflected in narrative comments. Themes that emerged included uncertainty whether telehealth has a role in EM, skepticism that telehealth required a specific curriculum (ie, that it was functionally identical to in-person care), and recognition that operational barriers to telehealth implementation limited efforts to teach it.

## DISCUSSION

Our data suggest that relatively few EM residency programs currently have telehealth education in place (7%, 5/67). The drivers of this seem to be multifactorial. Primary among these is the structural barrier to telehealth education. Many programs lack an existing telehealth platform or the institutional commitment to develop one. Developing an integrated clinical telehealth service is challenging and may require support outside the ED to actualize. In the absence of providing clinical telehealth services to patients (69% of respondents did not), it is perhaps unsurprising then that many residency faculty lack expertise in the area, further limiting efforts to educate residents. Recognizing these barriers, the majority of program directors (60%) without telehealth curricula reported interest in partnering with trusted national EM organizations to leverage outside expertise and circumvent operational hurdles to implementing clinical telehealth.

Our data also suggest that telehealth education faces an uphill struggle when it comes to its perceived value and importance in EM. Of the 67 program directors surveyed, 41 (61%) expressed the opinion that telehealth education is of only marginal importance to EM training. This attitude likely reflects separate threads that emerged in our objective and narrative data. As noted earlier, some program directors felt as if telehealth care was sufficiently similar to in-person care that it did not merit a separate curriculum. Others expressed their belief that the role of telehealth in EM was limited at baseline, with one program even underscoring that it was not an ABEM requirement. While most program directors noted faculty and infrastructural barriers to implementing a telehealth curriculum (65% and 50%, respectively), the most common reason was that they simply did not consider telehealth education to be a curricular priority (76%, 47/62). Educational time can be a zero-sum game in residency and, not surprisingly, program directors allocate time to what they deem to be most important (or required). With only 25% (17/67) of emergency departments even offering clinical telehealth services, it is understandable how this combination of skepticism about and unfamiliarity with telehealth might lessen its perceived import to residency leadership.

Despite these barriers, there does appear to be some consensus regarding what competencies within telehealth would be most relevant to EM. Among the surveyed program directors, there was strong interest in curricula focused on patient safety and appropriate use of telehealth, communication skills, and data collection and assessment. These priorities were similar across programs with and without existing telehealth curricula and perhaps speak to an implicit recognition of the ways in which telehealth care differs from traditional bedside care. Among programs with existing telehealth curricula, there was an additional strong interest in obtaining external educational support to teach the ethical and legal considerations of telehealth, which is unsurprising given the ever-evolving medicolegal telehealth landscape.

## LIMITATIONS

Our survey study has several limitations. Most apparent is our low response rate (24%), which threatens the generalizability of our findings and conclusions. While existing survey literature suggests that low response rate does not necessarily entail non-response bias, we attempted to formally analyze the extent to which non-response bias might have affected our primary question as to the prevalence of existing telehealth curricula.^6^,^7^ To do so, we employed a wave analysis, which uses late survey respondents as a surrogate for non-respondents. We had one positive response in Wave 1 (1/42, 2.4%) and three in Wave 3 (3/11, 27.3%). A pairwise Fisher exact test was significant (*P* = .03), suggesting a component of non-response bias. (Of note, two of the three respondents in Wave 3 were affiliated with our home institution and were known to us prior to undertaking this landscape survey. While a wave analysis is intended to characterize the unknown group of non-respondents, our wave analysis may ironically have overstated the degree to which there are unaccounted-for telehealth education programs.) While we recognize this significant limitation, our results are consistent with what little data exist on this topic, and the general uniformity of our data (ie, telehealth’s relative absence) suggests to us it is unlikely that our survey missed large pockets of existing telehealth education.^8^ We also recognize that there may be inaccuracies embedded within our reported data. While program directors may be expert when it comes to their educational curricula, they may lack insight into larger departmental or institutional forces that pertain to telehealth implementation. Thus, some of the reported barriers and challenges to telehealth education may be perceived as opposed to substantive.

## CONCLUSION

Our data suggest that most EM residency programs in the U.S. have not adopted AAMC recommendations and do not have a formal telehealth curriculum. Among those few programs that do teach telehealth, their curriculum is driven by lectures and shiftwork, with a focus on patient safety, digital assessment, and communication skills. Among programs without telehealth curricula, we identified several barriers to its implementation, including limited access to clinical telehealth services, a lack of faculty expertise in the subject, insufficient time in existing residency curricula, and a perceived lack of importance among program directors. Considering these barriers, it remains to be seen how residency programs will meet the proposed ACGME requirement of a “structured telemedicine experience.” Our data suggest one possible avenue is the use of asynchronous content developed by trusted national EM organizations. More work will be required to understand the future and relevance of telehealth in EM residency education.

## Supplementary Information



## Figures and Tables

**Table 1:** Demographics of emergency medicine residencies whose program directors responded to a survey regarding the prevalence of (and barriers to) formal telehealth curricula.

	Overall, N = 67	Has curriculum, n = 5	No curriculum, n = 62
Residency type			
University based	33 (49%)	4 (80%)	29 (47%)
Community based, university affiliated	26 (39%)	1 (20%)	25 (40%)
Community based, non-affiliated	5 (7.5%)	0 (0%)	5 (8.1%)
Hybrid	1 (1.5%)	0 (0%)	1 (1.6%)
Not reported	4 (6.0%)	0 (0%)	4 (6.5%)
Patient demographic(s) served			
Rural	16 (24%)	1 (20%)	15 (24%)
Urban	44 (66%)	4 (80%)	40 (65%)
Suburban	31 (46%)	0 (0%)	31 (50%)
Not reported	4 (6.0%)	0 (0%)	4 (6.5%)
What is the length of your program?			
3 years	47 (70%)	4 (80%)	43 (69%)
4 years	16 (24%)	1 (20%)	15 (24%)
Not reported	4 (6.0%)	0 (0%)	4 (6.5%)
How many residents does your program have in each class?			
5–10	18 (27%)	1 (20%)	17 (27%)
11–15	33 (49%)	2 (40%)	31 (50%)
16–20	8 (12%)	2 (40%)	6 (9.7%)
21–25	2 (3.0%)	0 (0%)	2 (3.2%)
> 25	2 (3.0%)	0 (0%)	2 (3.2%)
Not reported	4 (6.0%)	0 (0%)	4 (6.5%)
In what region is your residency program?			
South	20 (30%)	0 (0%)	20 (32%)
Northeast	26 (39%)	3 (60%)	23 (37%)
Midwest	11 (16%)	2 (40%)	9 (15%)
West	6 (9.0%)	0 (0%)	6 (9.7%)
Not reported	4 (6.0%)	0 (0%)	4 (6.5%)
What is your department’s annual patient volume?			
20,000–40,000	3 (4.5%)	1 (20%)	2 (3.2%)
41,000–60,000	5 (7.5%)	0 (0%)	5 (8.1%)
61,000–80,000	11 (16%)	1 (20%)	10 (16%)
81,000–100,000	21 (31%)	0 (0%)	21 (34%)
> 100,000	23 (34%)	3 (60%)	20 (32%)
Not reported	4 (6.0%)	0 (0%)	4 (6.5%)
Does your department or division of emergency medicine offer clinical telehealth services?			
No	46 (69%)	1 (20%)	45 (73%)
Yes	17 (25%)	4 (80%)	13 (21%)
Not reported	4 (6.0%)	0 (0%)	4 (6.5%)

**Table 2:** Characteristics of existing telehealth curricula in U.S. emergency medicine residencies derived from a survey of program directors.

Which forms of telehealth do you currently address in your curriculum?	

Real-time telehealth	4 (80%)
Mobile health	2 (40%)
Physician-to-physician consultations	2 (40%)
Asynchronous store and forward	1 (20%)
Remote patient monitoring	1 (20%)

How is the curricular content delivered?	

Live lectures	4 (80%)
Mandatory shifts	3 (60%)
Simulation	2 (40%)
Elective shifts	1 (20%)

Of the six telehealth competencies outlined by the AAMC, which are addressed in the learning objectives of your telehealth curriculum?	

Data collection and assessment via telehealth	4 (80%)
Patient safety and appropriate use of telehealth	4 (80%)
Communication via telehealth	3 (60%)
Ethical practices and legal requirements for telehealth	3 (60%)
Technology for telehealth	3 (60%)
Access and equity in telehealth	2 (40%)

For which of the telehealth competencies would your program most benefit by having access to high-quality, externally created educational content? (Select all that apply.)	

Ethical practices and legal requirements for telehealth	4 (80%)
Communication via telehealth	3 (60%)
Access and equity in telehealth	2 (40%)
Data collection and assessment via telehealth	2 (40%)
Technology for telehealth	2 (40%)
Patient safety and appropriate use of telehealth	2 (40%)

Which form(s) of external curricular content would best help you deliver (or augment) telehealth education at your program?	

Simulation	3 (60%)
Asynchronous content created by a third-party national EM organization (eg, ACEP/SAEM/AAEM)	2 (40%)
Live lectures	2 (40%)
Asynchronous content created by a third-party commercial entity	1 (20%)
Asynchronous content created by your institution	1 (20%)
Mandatory shifts	1 (20%)

Note: Response categories are not mutually exclusive. Response percentages represent proportion of sample with response and do not necessarily sum to 100%.

*AAMC*, Association of American Medical Colleges; *ACEP*, American College of Emergency Physicians; *SAEM*, Society for Academic Emergency Medicine; *AAEM*, American Academy of Emergency Medicine.

**Table 3:** Barriers to formal telehealth curricula noted by program directors who responded to a survey regarding the prevalence of (and barriers to) formal telehealth curricula in U.S. emergency medicine residency programs.

What has prevented your program from implementing a formal telehealth curriculum?	

Do not feel this is a curricular priority at this time	47 (76%)
There are insufficient faculty with telehealth expertise to run the curriculum	40 (65%)
There are insufficient faculty with telehealth expertise to develop the curriculum	38 (61%)
There is insufficient infrastructure to run the curriculum	31 (50%)
Did not realize this was an AAMC recommendation	25 (40%)
There is insufficient time or resources	25 (40%)
There is no time in the residency curriculum to add this domain	25 (40%)
Department does not use telehealth	2 (3.2%)
Current reimbursement model for telehealth is not viable	1 (1.6%)
Low telehealth patient volume	1 (1.6%)
Just started a telehealth program	1 (1.6%)
Telehealth education available as an elective only	1 (1.6%)
Not an ABEM requirement	1 (1.6%)

Were you to create a telehealth curriculum for your residency, which of the following six competencies (as outlined by the AAMC) would you prioritize?	

Patient safety and appropriate use of telehealth	40 (65%)
Communication via telehealth	36 (58%)
Ethical practices and legal requirements for telehealth	32 (52%)
Data collection and assessment via telehealth	31 (50%)
Access and equity in telehealth	29 (47%)
Technology for telehealth	19 (31%)

Which form(s) of content would best help you deliver a formal telehealth curriculum at your program?	

Asynchronous content created by a third-party national EM organization (eg, ACEP/SAEM/AAEM)	37 (60%)
Simulation	24 (39%)
Elective shifts	23 (37%)
Live lectures	17 (27%)
Asynchronous content created by your institution	16 (26%)
Mandatory shifts	11 (18%)
Asynchronous content created by a third-party commercial entity	10 (16%)
Asynchronous content that meets criteria for conference credit	1 (1.6%)
It would be part of the existing clinical curriculum	1 (1.6%)

Note: Response categories are not mutually exclusive. Response percentages represent proportion of sample with response and do not necessarily sum to 100%.

*AAMC*, Association of American Medical Colleges; *EM*, emergency medicine; *ACEP*, American College of Emergency Physicians; *SAEM*, Society for Academic Emergency Medicine; *AAEM*, American Academy of Emergency Medicine.
