# Supplementary material for: National Survey of Telemedicine Curricula Among Emergency Medicine Residencies
Source: West J Emerg Med. 2026 May 3;27(3):540–7. doi: 10.5811/westjem.52946 (PMC13246168; doi:10.5811/westjem.52946)
Supplement: Supplementary file 1 [file wjem-27-540-s001.pdf]

Telehealth Education and Learning Environment (TELE) Survey

Survey Flow

Block: Yes/No Differentiation (2 Questions)
Standard: Yes block (5 Questions)
Standard: No block (3 Questions)
Standard: Demographics (9 Questions)
Standard: Block 4 (1 Question)

Page Break

Start of Block: Yes/No Differentiation

Page Break

Q1 Does your residency have a formal Telehealth curriculum?

○ Yes (1)

○ No (2)

Page Break

Q26 In your opinion, how important will Telehealth education be to EM residency training in the future?

	Not at all important (1)	Slightly important (2)	Moderately important (3)	Very important (4)	Extremely important (5)
Please choose one of the following options: (1)	○	○	○	○	○

End of Block: Yes/No Differentiation

Start of Block: Yes block

Display this question:

If Does your residency have a formal Telehealth curriculum? = Yes

Q9 Which forms of Telehealth do you currently address in your curriculum? (Select all that apply)

- ☐ **Real-time Telehealth** [*Real-time Telehealth means real-time care between a doctor and patient at any time from anywhere. Real-time Telehealth includes any two-way communications involving a connection via video conferencing and phone consultations, that connect providers and patients.*] (1)

- ☐ **Asynchronous store and forward** [*Store-and-forward Telehealth makes patient records and medical data more accessible across long distances. Medical data, including images, test results, bio-signals, lab reports, and substantial documents can be acquired and transmitted (stored-and-forwarded) across distances.*] (2)

- ☐ **Physician to physician consultations** [*Being able to consult another physician via phone or video*] (3)

- ☐ **Remote patient monitoring** [*Remote patient monitoring technology shares patient medical data regularly with the patient's care team. This technology transmits the information automatically, leading to active ongoing care monitoring in a more passive data collection process.*] (4)

- ☐ **Mobile health** [*Consumer technologies like smartphone and tablet apps that enable consumers to capture their own health data*] (5)

- ☐ **Other** [*please describe*] (6) ____

- ☐ I am unable to answer this question (7)

Page Break

Display this question:

If Does your residency have a formal Telehealth curriculum? = Yes

Q10 How is the curricular content delivered? (Select all that apply)

- ☐ Asynchronous content created by your institution (1)
- ☐ Asynchronous content created by a third party commercial entity (2)
- ☐ Asynchronous content created by a third party national EM organization (e.g. ACEP/SAEM/AAEM) (3)
- ☐ Live lectures (4)
- ☐ Elective Shifts (5)
- ☐ Mandatory Shifts (6)
- ☐ Simulation (7)
- ☐ Other [please describe] (8) ____
- ☐ I am unable to answer this question (9)

Page Break

Display this question:

If Does your residency have a formal Telehealth curriculum? = Yes

Q13 Of the six Telehealth competencies outlined by AAMC, which are addressed in the learning objectives of your Telehealth curriculum? Examples in parentheses below are at the "Recent Residency Graduate" level as defined by the AAMC. (Select all that apply)

▢ **Patient Safety and Appropriate Use of Telehealth** (*Adapts practice in context of the limitations and benefits of Telehealth. Evaluates and remedies patient and practice barriers to incorporating Telehealth into care. Demonstrates understanding of all roles and works as a team member when practicing Telehealth regardless of modality. Prepares for and escalates care when patient safety is at risk during a Telehealth encounter.*) (1)

▢ **Access and Equity in Telehealth** (*Describes and mitigates one's own implicit and explicit biases during Telehealth encounters. Leverages technology to promote health equity and mitigate gaps in access to care. Accommodates the patient's needs, preferences, and potential cultural, social, physical, cognitive, linguistic and communication barriers to technology use.*) (2)

▢ **Communication via Telehealth** (*Develops an effective rapport with patients via video visits, attending to eye contact, tone, body language, and nonverbal cues. Establishes therapeutic relationships and environments during video visits, such as attending to disruptions related to privacy, lighting, sound, and attire. Determines situations in which patients' social supports and health care providers should be incorporated into Telehealth interactions, with the patients' consent, to provide optimal care.*) (3)

▢ **Data Collection and Assessment via Telehealth** (*Obtains history and incorporates the information into differential diagnosis and the management plan. Conducts appropriate physical examination and collects relevant data on clinical status, including guiding the patient and/or tele-presenter. Incorporates patient-generated data into the clinical assessment and treatment plan while understanding data limitations and adapting accordingly.*) (4)

▢ **Technology for Telehealth** (*Identifies and is able to use the equipment needed for the intended service at both originating and distant sites. Practices with a wide range of evidence-based technologies, including patient-owned devices, and understands limitations. Demonstrates how to troubleshoot basic technology failures and optimize settings with the technology being used.*) (5)

▢ **Ethical Practices and Legal Requirements for Telehealth** (*Complies with legal and privacy regulations for Telehealth at the local, state, and federal levels. Obtains informed consent and maintains privacy. Identifies and supports solutions that mitigate ethical problems and adhere to professional requirements in Telehealth. Explains and discloses potential conflicts of interest to patients in use of Telehealth.*) (6)

▢ I am unable to answer this question (7)

Page Break

Display this question:

If Does your residency have a formal Telehealth curriculum? = Yes

Carry Forward All Choices - Displayed & Hidden from "Of the six Telehealth competencies outlined by AAMC, which are addressed in the learning objectives of your Telehealth curriculum? Examples in parentheses below are at the "Recent Residency Graduate" level as defined by the AAMC. (Select all that apply)"

Q16 For which of the Telehealth competencies would your program most benefit by having access to high-quality, externally-created educational content? (Select all that that apply)

▢ **Patient Safety and Appropriate Use of Telehealth** (*Adapts practice in context of the limitations and benefits of Telehealth. Evaluates and remedies patient and practice barriers to incorporating Telehealth into care. Demonstrates understanding of all roles and works as a team member when practicing Telehealth regardless of modality. Prepares for and escalates care when patient safety is at risk during a Telehealth encounter.*) (1)

▢ **Access and Equity in Telehealth** (*Describes and mitigates one's own implicit and explicit biases during Telehealth encounters. Leverages technology to promote health equity and mitigate gaps in access to care. Accommodates the patient's needs, preferences, and potential cultural, social, physical, cognitive, linguistic and communication barriers to technology use.*) (2)

▢ **Communication via Telehealth** (*Develops an effective rapport with patients via video visits, attending to eye contact, tone, body language, and nonverbal cues. Establishes therapeutic relationships and environments during video visits, such as attending to disruptions related to privacy, lighting, sound, and attire. Determines situations in which patients' social supports and health care providers should be incorporated into Telehealth interactions, with the patients' consent, to provide optimal care.*) (3)

▢ **Data Collection and Assessment via Telehealth** (*Obtains history and incorporates the information into differential diagnosis and the management plan. Conducts appropriate physical examination and collects relevant data on clinical status, including guiding the patient and/or tele-presenter. Incorporates patient-generated data into the clinical assessment and treatment plan while understanding data limitations and adapting accordingly.*) (4)

▢ **Technology for Telehealth** (*Identifies and is able to use the equipment needed for the intended service at both originating and distant sites. Practices with a wide range of evidence-based technologies, including patient-owned devices, and understands limitations. Demonstrates how to troubleshoot basic technology failures and optimize settings with the technology being used.*) (5)

▢ **Ethical Practices and Legal Requirements for Telehealth** (*Complies with legal and privacy regulations for Telehealth at the local, state, and federal levels. Obtains informed consent and maintains privacy. Identifies and supports solutions that mitigate ethical problems and adhere to professional requirements in Telehealth. Explains and discloses potential conflicts of interest to patients in use of Telehealth.*) (6)

▢ I am unable to answer this question (7)

Page Break

Display this question:

If Does your residency have a formal Telehealth curriculum? = Yes

Q14 Which form(s) of external curricular content would best help you deliver (or augment) Telehealth education at your program? (Select all that apply)

- ▢ Asynchronous content created by your institution (1)
- ▢ Asynchronous content created by a third party commercial entity (2)
- ▢ Asynchronous content created by a third party national EM organization (e.g. ACEP/SAEM/AAEM) (3)
- ▢ Live lectures (4)
- ▢ Elective Shifts (5)
- ▢ Mandatory Shifts (6)
- ▢ Simulation (7)
- ▢ Other [please describe] (8) ____
- ▢ I am unable to answer this question (9)

End of Block: Yes block

Start of Block: No block

Display this question:

If Does your residency have a formal Telehealth curriculum? = No

Q11 What has prevented your program from implementing a formal Telehealth curriculum? (Select all that apply)

- ▢ Did not realize this was AAMC recommendation (1)
- ▢ Do not feel this is a curricular priority at this time (2)
- ▢ There is no time in the residency curriculum to add this domain (3)
- ▢ There are insufficient faculty with Telehealth expertise to develop the curriculum (4)
- ▢ There are insufficient faculty with Telehealth expertise to run the curriculum (5)
- ▢ There is insufficient infrastructure to run the curriculum (e.g. no existing patient portal, EMR does not support Telehealth, no webcams, et cetera) (6)
- ▢ There is insufficient time or resources (e.g. faculty compensation or administrative support) (7)
- ▢ Other [please describe] (8) ____
- ▢ I am unable to answer this question (9)

Page Break

Display this question:

If Does your residency have a formal Telehealth curriculum? = No

Q23 Were you to create a Telehealth curriculum for your residency, which of the following six competencies (as outlined by the AAMC) would you prioritize? Examples in parentheses below are at the "Recent Residency Graduate" level as defined by the AAMC. (Select all that apply)

▢ **Patient Safety and Appropriate Use of Telehealth** (*(Adapts practice in context of the limitations and benefits of Telehealth. Evaluates and remedies patient and practice barriers to incorporating Telehealth into care. Demonstrates understanding of all roles and works as a team member when practicing Telehealth regardless of modality. Prepares for and escalates care when patient safety is at risk during a Telehealth encounter).*) (1)

▢ **Access and Equity in Telehealth** (*Describes and mitigates one's own implicit and explicit biases during Telehealth encounters. Leverages technology to promote health equity and mitigate gaps in access to care. Accommodates the patient's needs, preferences, and potential cultural, social, physical, cognitive, linguistic and communication barriers to technology use.*) (2)

▢ **Communication via Telehealth** (*Develops an effective rapport with patients via video visits, attending to eye contact, tone, body language, and nonverbal cues. Establishes therapeutic relationships and environments during video visits. Determines situations in which patient's social supports and health care providers should be incorporated to provide optimal care.*) (3)

▢ **Data Collection and Assessment via Telehealth** (*Obtains history and incorporates the information into differential diagnosis and the management plan. Conducts appropriate physical examination and collects relevant data on clinical status, including guiding the patient and/or tele-presenter. Incorporates patient-generated data into the clinical assessment and treatment plan while understanding data limitations and adapting accordingly.*) (4)

▢ **Technology for Telehealth** (*Identifies and is able to use the equipment needed for the intended service at both originating and distant sites. Practices with a wide range of evidence-based technologies, including patient-owned devices, and understands limitations. Demonstrates how to troubleshoot basic technology failures and optimize settings with the technology being used.*) (5)

▢ **Ethical Practices and Legal Requirements for Telehealth** (*Complies with legal and privacy regulations for Telehealth at the local, state, and federal levels. Obtains informed consent and maintains privacy. Identifies and supports solutions that mitigate ethical problems and adhere to professional requirements in Telehealth. Explains and discloses potential conflicts of interest to patients in use of Telehealth.*) (6)

▢ I am unable to answer this question (7)

Page Break

Display this question:

If Does your residency have a formal Telehealth curriculum? = No

Q21 Of the following, which form(s) of content would best help you deliver a formal Telehealth curriculum at your program? (Select all that apply)

▢ Asynchronous content created by your institution (1)

▢ Asynchronous content created by a third party commercial entity (2)

▢ Asynchronous content created by a third party national EM organization (e.g. ACEP/SAEM/AAEM) (3)

▢ Live lectures (4)

▢ Elective Shifts (5)

▢ Mandatory Shifts (6)

▢ Simulation (7)

▢ Other [please describe] (8) ____

▢ I am unable to answer this question (9)

End of Block: No block

Start of Block: Demographics

Q31 Please select your residency program. (This information is only used to identify which programs to contact for survey completion; there will be no identifiers used in the reporting of survey results.)

▼ I would prefer not to answer (1) ... Other (284)

Q2 What is the length of your program?

- ◯ 3 years (1)
- ◯ 4 years (2)
- ◯ Other [please describe] (3) ____

Q3 How many residents (on average) does your program have in each class?

- ◯ Fewer than 5 (1)
- ◯ 5-10 (2)
- ◯ 11-15 (3)
- ◯ 16-20 (4)
- ◯ 21-25 (5)
- ◯ Greater than 25 (6)

Q4 How many full-time faculty (in total) are there in your program?

- ◯ Fewer than 10 (1)
- ◯ 10-25 (2)
- ◯ 26-50 (3)
- ◯ 51-75 (4)
- ◯ 76-100 (5)
- ◯ Greater than 100 (6)

Q5 What patient demographic does your program serve? (Select all that apply)

- ▢ Rural (1)
- ▢ Suburban (2)
- ▢ Urban (3)

Q6 Which of the following best describes your residency program? (Select all that apply)

- ▢ University based (1)
- ▢ Community based, university affiliated (2)
- ▢ Community based, nonaffiliated (3)
- ▢ Military (4)
- ▢ Other [please describe] (5) ____

Q7 In what region is your residency program?

- ○ South (1)
- ○ Northeast (2)
- ○ Midwest (3)
- ○ West (4)
- ○ Other [please describe] (5) ____

Q8 What is the annual patient volume at your program's Emergency Department(s)?

- ○ Fewer than 20,000 (1)
- ○ 20,000-40,000 (2)
- ○ 41,000-60,000 (3)
- ○ 61,000-80,000 (4)
- ○ 81,000-100,000 (5)
- ○ Greater than 100,000 (6)

Q23 Does your Department or Division of Emergency Medicine offer clinical Telehealth services at this time?

- ○ Yes (1)
- ○ No (2)

End of Block: Demographics

Start of Block: Block 4

Q27 Thank you for taking the time to complete our survey. This is the last question of the survey. Please use this space to write out any additional comments you may have regarding Telehealth Education. If you do not have any additional comments, please click the arrow at the bottom to submit your survey response.

____

____

____

____

____

End of Block: Block 4
